# Understanding the phyto-interaction of heavy metal oxide bulk and nanoparticles: evaluation of seed germination, growth, bioaccumulation, and metallothionein production†

**DOI:** 10.1039/c8ra09305a

**Published:** 2019-02-01

**Authors:** Bilal Ahmed, Asfa Rizvi, Almas Zaidi, Mohammad Saghir Khan, Javed Musarrat

**Affiliations:** Department of Agricultural Microbiology, Faculty of Agricultural Sciences, Aligarh Muslim University Aligarh 202002 U.P. India bilalahmed.amu@gmail.com bilalahmed.rs@amu.ac.in +91-9045836145

## Abstract

The fast-growing use of nano-based products without proper care has led to a major public health concern. Nanomaterials contaminating the environment pose serious threat to the productivity of plants and *via* food chain to human health. Realizing these, four vegetable crops, radish, cucumber, tomato, and alfalfa, were exposed to varying concentrations of heavy metal oxide (TiO_2_, ZnO, Al_2_O_3_ and CuO) submicron or bulk (BPs) and nanoparticles (NPs) to assess their impact on relative seed germination (RSG), seed surface adsorption, root/shoot tolerance index (RTI/STI), bioaccumulation, and metallothioneins (MTs) production. The results revealed a clear inhibition of RSG, RTI, and STI, which, however, varied between species of metal-specific nanoparticles and plants. SEM and EDX analyses showed significant adsorption of MONP agglomerates on seed surfaces. The concentration of metals detected by EDX differed among vegetables. Among the metals, Al, Cu, Ti, and Zn were found maximum in alfalfa (12.46%), tomato (23.2%), cucumber (6.32%) and radish (21.74%). Of the four metal oxides, ZnO was found most inhibitory to all vegetables and was followed by CuO. The absorption/accumulation of undesirable levels of MONPs in seeds and seedlings differed with variation in dose rates, and was found to be maximum (1748–2254 μg g^−1^ dry weight) in ZnO-NPs application. Among MONPs, the uptake of TiO_2_ was minimum (2 to 140 μg g^−1^) in radish seedlings. The concentration of MTs induced by ZnO-NPs, ZnO-BPs, and CuO-NPs ranged between 52 and 136 μ mol MTs g^−1^ FW in vegetal organs. Conclusively, the present findings indicated that both the nanosize and chemical composition of MONPs are equally dangerous for vegetable production. Hence, the accumulation of MONPs, specifically ZnO and CuO, in edible plant organs in reasonable amounts poses a potential environmental risk which, however, requires urgent attention to circumvent such toxic problems.

## Abstract 2

Phyto-interaction of heavy metal oxide nano and bulk particles with agriculturally important crops.

## Graphical abstract

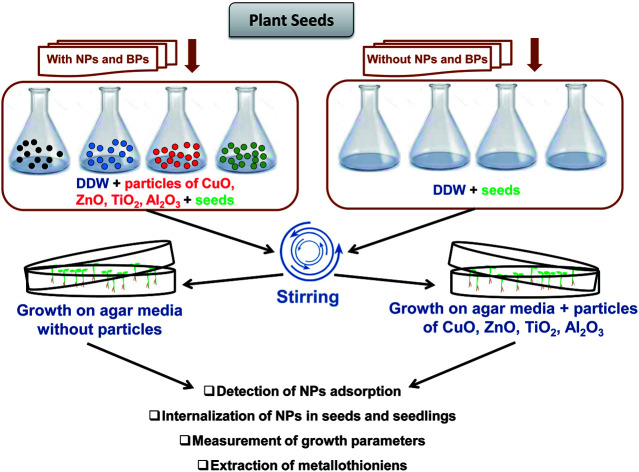

## 1. Introduction

Nano-technological advancements on the one hand have great potential in many environmental and industrial applications, while on the other hand they raise serious concerns over the use of NPs due to environmental problems.^1^ Among various NPs, metal oxide nanoparticles (MONPs) for example, ZnO, CuO, TiO_2_, Al_2_O_3_, ZrO_2_, Fe_2_O_3_, Ag_2_O, CeO_2_ and NiO are widely used in many industries such as cosmetics, energy production, paints, textiles, and rocket fuels, and in biomedical applications.^1,2^ Apart from these, MONPs have also been applied in agriculture practices as nano-fertilizers and in protecting plants from pathogens.^3,4^ Due to the ever-increasing demands, it is likely that the production of MONPs which was just 0.27 million tons in 2012 will increase to 1.663 million tons by 2020.^5^ Of the total production, 8–28%, 0.4–7.0%, and 0.1–1.5% MONPs are expected to accumulate in the soil system, water and atmosphere, respectively, after production, application, and discharge.^5^ Once deposited in soil either through nano-products such as fertilizers, insecticide, and pesticides^1,4^ or from other sources, the MONPs may become toxic to bacteria, plants, animal, and human cells.^6–9^ Despite these, the understanding on lethality of MONPs is still limited and hence, requires special attention to better understand the consequences of MONPs on crop production.^10^ However, in this context, a very few attempts have been made to assess the biological impacts of NPs in controlled laboratory conditions with single species of model organisms, which are essential to elucidate the interaction mechanism of NPs.^11^

Indeed, plants are critical for the sustenance of the ecosystem, and due to the direct association of roots with soil ecosystem, plants come in direct contact with soil constituents either present naturally or deposited anthropogenically.^12^ Among anthropogenic materials, the MONPs penetrate plant cells either through the process of endocytosis or by other transport systems and are bioaccumulated inside plant tissues.^5^ However, the uptake, translocation, and bioaccumulation of MONPs in plants depend upon the size, chemical composition and shape of the MONPs and plant anatomy.^13,14^ Following accumulation, they cause morpho-biochemical changes in plants.^10^ For example, ZnO-NPs, TiO_2_-NPs, Al_2_O_3_-NPs, CuO-NPs, NiO-NPs, CdO-NPs, and Fe_2_O_3_-NPs have been found accumulated in plant tissues and are reported to be toxic to major agriculture crops such as soybean, corn, cucumber, tomato, wheat, maize, mung, bean, chickpea, spinach, and barley.^1,5,15^ However, there are only few reports on the assessment of the effect of a single type of MONPs on different plant species under identical growth environment. For instance, García-Gómez *et al.* reported the comparative phytotoxicity of ZnO-NPs on nine crops grown in calcareous and acidic soil.^10^ The study suggested that plant species and soil pH were key factors affecting the availability of Zn and toxicity of ZnO-NPs. In yet another study, the toxicity of various MONPs such as CuO, Al_2_O_3_, MnO, Fe_3_O_4_, ZnO, and TiO_2_ was tested against germinating seeds of *Sinapis alba*.^13^ Among MONPs, Al_2_O_3_, MnO, Fe_3_O_4_, and TiO_2_ did not affect seed germination, while ZnO-NPs and CuO-NPs inhibited germination in a dose-dependent manner. Interestingly, like metals, the varying impact of NPs on different plant species also depends on the size, concentration, duration of exposure, plant genotypes and experimental conditions.^16^ The different thresholds of NP toxicity in plants in different experimental setups may further complicate the classification of plants into tolerant or sensitive groups. This situation demands the examination of nano-phytotoxicity among different plant species. Therefore, in our study we exposed four vegetable crops including radish (*Raphanus sativus*), cucumber (*Cucumis sativus*), tomato (*Solanum lycopersicon*), and alfalfa (*Medicago sativa*) to varying levels of four MONPs (ZnO, CuO, TiO_2_, and Al_2_O_3_ NPs) along with their bulk counterparts (BPs) to explain the following – (i) adsorption of heavy MONPs on seeds and relative seed germination, (ii) root and shoot tolerance index, (iii) uptake of metal by seeds and seedlings, and (iv) MTs production under stress. The four vegetables used in this study were intentionally chosen to assess the phyto-interaction of four MONPs, ZnO, Al_2_O_3_, TiO_2_, and CuO largely due to their high demand in human dietary system. Also, these vegetables, are reported to respond well to NPs such as Ag, Ni, CeO_2_, ZnO, and Fe_3_O_4_.^1,15^ Apart from these, submicron- and nano-forms of metal oxides of Cu, Al, Zn, and Ti were selected keeping in mind their use in various industrial products including explosives, alloys, drug delivery tools, personal care products, catalysts, sensors, semiconductor devices, batteries, microelectronics, antimicrobial coatings, textiles, paints and food containers.^2,4,5,13^ When discharged into the environment without proper treatment, such MONPs destruct the very sustainability of agro-eco systems.^15^ Due to these and several other reasons, four MONPs were selected to evaluate their nano–phyto-interaction activity against popularly grown vegetables worldwide.

## 2. Materials and methods

### 2.1 Nanoparticles, bulk particles and their characterization

The MONPs namely, ZnO-NPs (product code 2640103), CuO-NPs (product code 2040263), TiO_2_-NPs (product code 28954), and Al_2_O_3_-NPs (product code 75364) purchased from Sisco Research Laboratories (Mumbai, India) were thoroughly characterized (please see ESI†). Before application, NPs were ultrasonicated at 60% amplitude for 30 min in double distilled water (DDW) in an ice bath.

### 2.2 Seedling growth and exposure conditions

**Fig. 1:**
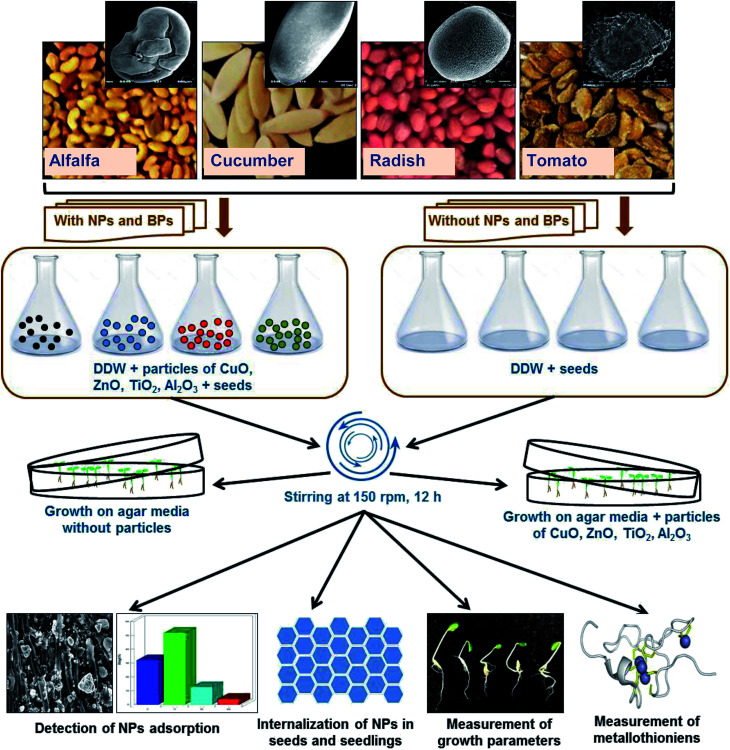
Experimental outline of the assessment of MONPs in alfalfa, cucumber, radish, and tomato plants.

The outline of the assessment of heavy MONPs in agricultural crops is presented in Fig. 1. Healthy seeds of *R. sativus* var. Meena early, *C. sativus* var. Karina, *S. lycopersicon* var. NP-7715 and *M. sativa* var. Chetak S-244 were properly sterilized using 2% solution of sodium hypochlorite (NaOCl) for 10 min. Seeds were exposed to NPs and BPs of TiO_2_, ZnO, Al_2_O_3_, and CuO in two sets of experiments – (i) seeds (*N* = 30) from each plant species were soaked in 0.05, 0.5, 2, and 5 mg ml^−1^ of NPs and BPs prepared in DDW for 12 h and kept on a rotatory shaker (150 rpm) at 25 ± 2 °C. Subsequently, seeds were rinsed with DDW and transferred to semi solid agar (0.4%) medium mixed with modified composition of 1/4 Hoagland nutrient medium^12^ maintaining ≥1 cm distance among seeds. (ii) Sterilized seeds (*N* = 10) were directly transferred on to semi-solid (0.4%) Hoagland 1/4 nutrient agar media in Petri dishes (100 × 20 mm) supplemented with 0.05, 0.5, 2, and 5 mg ml^−1^ of NPs/BPs of TiO_2_, ZnO, Al_2_O_3_, and CuO. Petri dishes containing seeds were then placed in a growth chamber at 25 ± 2 °C for germination and allowed to grow further. Each individual experiment was repeated three times.

### 2.3 Adsorption of NPs on plant seeds

Localized NPs of TiO_2_, ZnO, Al_2_O_3_, and CuO on seed surface were analyzed by SEM and EDX spectroscopy. Seeds dipped in MONPs were continuously stirred at 150 rpm in order to avoid the sedimentation of MONPs at the bottom of the flasks. Seeds exposed to 2 mg ml^−1^ concentration of each of the NPs were rinsed with sterile DDW and fixed in 2.5% glutaraldehyde and 2% paraformaldehyde in 0.1 M sodium phosphate buffer (pH 7.2) for 2 h at room temperature and at 4 °C for 6 h with intermittent vortexing. After three rinses with 0.1 M sodium phosphate buffer (pH 7.2), the seeds were dehydrated in a gradient of ethanol ranging from 30% to 100% for 10 min in each. The dehydrated seeds were then processed for SEM and EDX analysis (please see ESI†).

### 2.4 Analysis of growth parameters

Percent seed germination and root and shoot tolerance index (%) were measured for all replicates in dark at 25 ± 2 °C after 4 days of *R. sativus* growth, 7 days of *M. sativa* growth, and after 6 days each of *C. sativus* and *S. lycopersicon* growth. A seed was considered germinated after the emergence of plumule or radicles from the seed coat. From the data obtained, three calculations were made:1
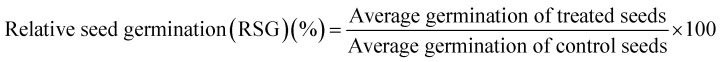
2
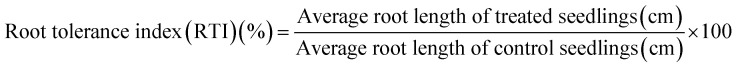
3
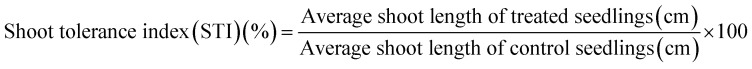


The RTI and STI were measured after 4 days of seed germination.

### 2.5 Internalization of metal in plant seeds and seedlings

To estimate the amount of TiO_2_, ZnO, Al_2_O_3_, and CuO NPs deposited in *R. sativus*, *C. sativus*, *S. lycopersicon*, and *M. sativa*, seeds (after soaking for 12 h) and germinated seedlings from treated and control groups were allowed to dry at 60 °C for 24 h. The dried biomass was further processed (please see ESI†).

### 2.6 Extraction and measurement of metallothioneins

Sterilized seeds of radish, cucumber, tomato and alfalfa with and without soaking in NP solutions and grown in the absence and presence of 0.05, 0.5, 2, and 5 mg ml^−1^ of Al_2_O_3_-NPs, CuO-NPs, TiO_2_-NPs and ZnO-NPs, respectively, were incubated on soft agar medium for 10 days in dark at 25 °C and allowed to grow. Thereafter, the roots emerging from the experimental plants were detached and washed to remove adhering particles. The seedlings treated with 200 μM Cu^2+^ served as the positive control. A total of 0.5 g root tissue was crushed in extraction buffer (pH 8.0) containing 0.7 M sucrose, 0.5 M tris, 50 mM EDTA, 0.1 M KCl, and 1 mM phenylmethyl sulfonyl fluoride (PMSF). To this, β-mercaptoethanol (β-ME) was added just before the extraction process maintaining 0.01% β-ME in the extraction buffer. The tissues were sonicated for 2 min (30/30 seconds pulse on/off) at 40% amplitude in ice bath. The sonicated samples were centrifuged at 8000 rpm for 30 min at 4 °C to obtain a supernatant containing metallothionein. A total of 2 ml of the resulting supernatant was mixed with 2 ml pre-chilled ethanol and 80 μl chloroform and vortexed. The samples were centrifuged in cold (0–4 °C) at 5000 rpm for 5 min and three volumes of pre-chilled ethanol was added to the resulting supernatant and kept at −20 °C for at least 1 h. The pellet was spun again in cold (0–4 °C) at 5000 rpm for 5 min, and the pellets were washed with a mixture of ethanol : chloroform : homogenization buffer (0.7 M sucrose, 0.5 M tris, pH 8.0 containing β-ME 0.01%) in the ratio of 87 : 1 : 12. The pellets were air dried and re-suspended in 500 μl of 5 mM tris and 1 mM EDTA mixture at pH 7. To this fraction of MTs, 4.2 ml of 0.43 mM Ellman's reagent (5,5′-dithiobis-(2-nitrobenzoic acid)) in 0.2 M potassium phosphate buffer was added at pH 8 and incubated at room temperature for 30 min. The absorbance was recorded at 412 nm to estimate the concentration of reduced sulfhydryl (–SH). A standard linear curve of reduced glutathione was run in parallel from 13.33–133.3 μM at 412 nm (*r*^2^ = 0.98) (ESI Fig. 1†). The GSH containing one cysteine per molecule serves as reference for quantifying cysteines in protein. The amount of MTs in the samples was calculated assuming that 1 mol of MT contains 20 mol of cysteine. The MTs in the samples were measured using the standard curve of reduced glutathione (GSH).

### 2.7 Statistical analysis

All experiments were performed three times and statistical significance was calculated at 95% confidence limit (*P* ≤ 0.05). Microsoft Excel (2016) and Sigma plot 10.0 were used to prepare curves and graphs. Statistical analyses were performed using one-way analysis of variance (ANOVA) using statistical software Minitab 17 (Minitab Inc., State College PA, USA).

## 3. Results and discussion

### 3.1 Characterization of NPs and BPs

**Fig. 2:**
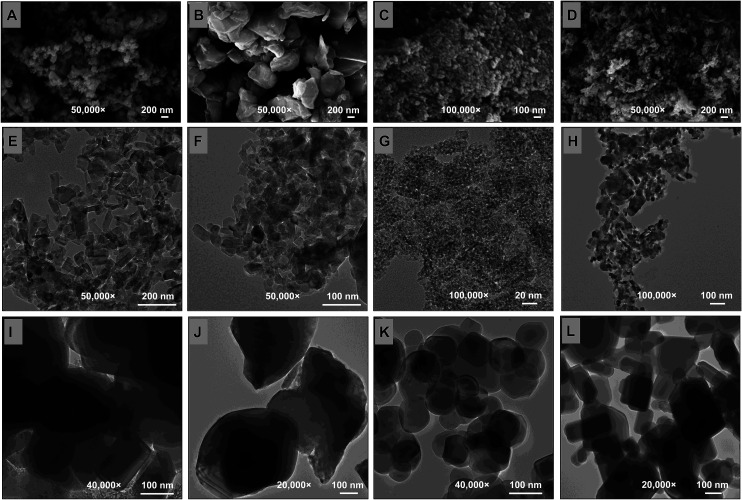
SEM (A–D) and TEM micrographs of NPs (E–H) and BPs (I–L): Al_2_O_3_-NPs (A and E), CuO-NPs (B and F), TiO_2_-NPs (C and G), ZnO-NPs (D and H), Al_2_O_3_-BPs (I), CuO-BPs (J), TiO_2_-BPs (K), and ZnO-BPs (L).

The surface morphology, average crystalline size and shape of MONPs were observed under XRD (ESI Fig. 2a–d†), FTIR (ESI Fig. 3a–d†), SEM (Fig. 2a–d), EDX (ESI Fig. 4a–d†), TEM (Fig. 2e–h), 2D-AFM (ESI Fig. 5a–d†) and 3D-AFM (ESI Fig. 5e–h†). The XRD pattern of MONPs ranged between 20 and 80 degree of 2*θ* values and were found similar to JCPDS file no. 77-2135, 45-0937, 78-2486, and 36-1451 for Al_2_O_3_-NPs (ESI Fig. 2a†), CuO-NPs (ESI Fig. 2b†), TiO_2_-NPs (ESI Fig. 2c†), and ZnO-NPs (ESI Fig. 2d†), respectively. The percent frequency size distribution of MONPs is shown in ESI Fig. 6a–d.† The SEM (Fig. 2a–d) and AFM (ESI Fig. 5a–h†) micrographs exhibited the topography and surface characteristic of MONPs, which indicated the presence of variable size aggregates of MONPs (Table 1). The FTIR data showed the characteristic metal–oxide (M–O) bond for all MONPs, which was found at 466, 533, 541, and 482 cm^−1^ for Al_2_O_3_-NPs, CuO-NPs, TiO_2_-NPs, and ZnO-NPs, respectively. The characteristics of the MONPs used in this study are presented in detail in Table 1. The average sizes of the BPs of Al_2_O_3_ (Fig. 2i), CuO (Fig. 2j), TiO_2_ (Fig. 2k), and ZnO (Fig. 2l) measured by TEM were 167, 586, 144, and 240 nm, respectively.

**Table 1:** Characteristics of heavy MONPs assessed in radish, cucumber, tomato, and alfalfa plants

Particulars	Al_2_O_3_-NPs	CuO-NPs	TiO_2_-NPs	ZnO-NPs
Size on packing (nm)	20–30	≈40	≈7	≈30
Assay, min (%)	99.9	99	95	99.9
Elemental% in EDX spectrum	Al (50.61), O (49.39)	Cu (76.7), O (23.3)	Ti (53.19), O (46.81)	Zn (78.92), O (21.08)
Morphology by SEM, AFM, and TEM	Spherical to lobular to short rods of variable length and diameter	Irregular individuals and aggregates with rough surface	Spherical with uniform size distribution	Pleomorphic, smaller to larger sized aggregates with some small thin sheets
Crystal size by XRD (nm)	28	18	4	24
Primary size by TEM (nm)	21.8 ± 8.7	18.4 ± 5.5	3.9 ± 0.9	34 ± 10
Secondary size by QLS (nm)	238 ± 4.6	194 ± 5.8	148 ± 8.4	248 ± 11.7
Zeta potential (mV)	+26.1 ± 1.7	−29.8 ± 2.1	+19.2 ± 2.3	−21 ± 0.9
Signal in IR spectrum (cm^−1^)	466	533	541	482

### 3.2 Adsorption of heavy MONPs on seeds

**Fig. 3:**
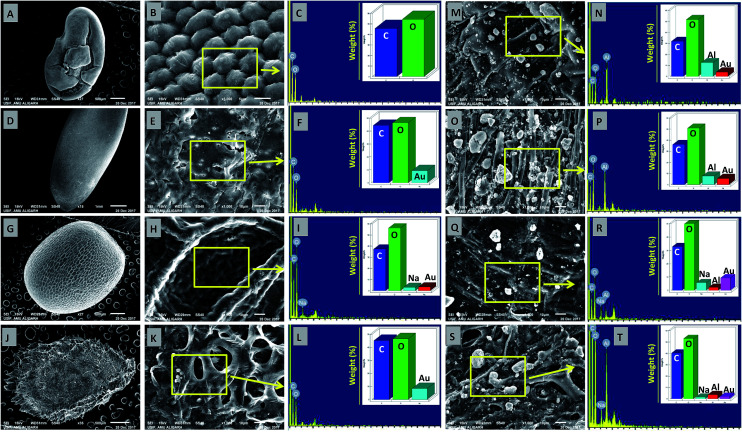
SEM and EDX-based analysis of untreated seeds of *M. sativa* (A–C), *C. sativus* (D–F), *R. sativus* (G–I), and *S. lycopersicon* (J–L). Panels (M–T) show the adsorption of Al_2_O_3_-NPs and their detection by EDX on the surface of *M. sativa* (M–N), *C. sativus* (O–P), *R. sativus* (Q–R), and *S. lycopersicon* (S–T) seeds after exposure to Al_2_O_3_-NPs in water for 12 h. EDX spectra showed metal peaks and weight percentage of different elements (N, P, R, and T).

**Fig. 4:**
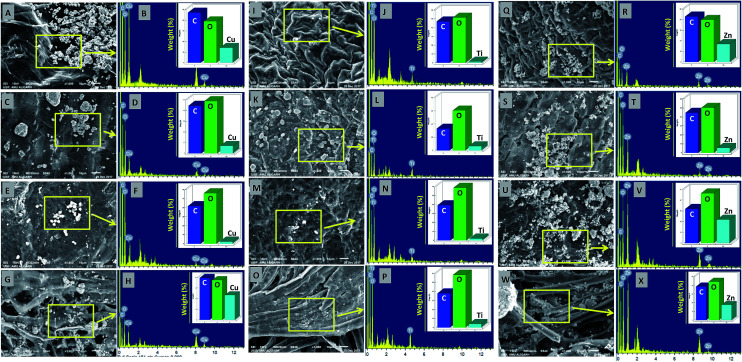
SEM and EDX-based analysis of CuO-NPs adsorption and their detection by EDX on the surface of *M. sativa* (A and B), *C. sativus* (C and D), *R. sativus* (E and F), and *S. lycopersicon* (G and H) seeds after exposure to CuO-NPs in water for 12 h. Panels (I–P) show the adsorption of TiO_2_-NPs and their detection by EDX on the surface of *M. sativa* (I and J), *C. sativus* (K and L), *R. sativus* (M and N), and *S. lycopersicon* (O and P), while panels (Q–X) show the adsorption of ZnO-NPs and their detection by EDX on the surface of *M. sativa* (Q and R), *C. sativus* (S and T), *R. sativus* (U and V), and *S. lycopersicon* (W and X). EDX spectra showed metal peaks and weight percentage of different elements.

The deposition of MONPs on the seed surface of radish, cucumber, tomato and alfalfa was detected by SEM equipped EDX after uniform shaking of seeds in an aqueous dispersion of MONPs. The data obtained are shown in Fig. 3 and 4. The SEM images represent the region of interest (ROI) on the seed surface for which the EDX spectrum was obtained from individual treatment. The weight percentage of different elements detected in EDX spectra is shown in the inset of each EDX spectrum as bars. Small and large aggregates of variable sizes were noticed on seed surfaces treated with Al_2_O_3_-NPs (Fig. 3m–t), CuO-NPs (Fig. 4a–h), TiO_2_-NPs (Fig. 4i–p), and ZnO-NPs (Fig. 4q–x). In contrast, the surface of seeds in the control group was clear and no heavy metal signal was obtained (Fig. 3a–l). Besides for C and O, signals for Au (Fig. 3i, n, p, r and t) and Na (Fig. 3i, r and t) were also noticed in some samples. The signals for Au might have come from the gold sputter coating done before SEM analysis, whereas, Na was one of the constituents of the phosphate buffer used for washing the seeds. The weight of metals expressed in percentage value as detected by EDX followed the order – (i) Al: 12.46, 7.66, 2.25, 3.59 (ii) Cu: 13.97, 6.90, 2.31, 23.20, (iii) Ti: 1.36, 6.32, 2.06, 3.84, and (iv) Zn: 17.13, 5.48, 21.74, 17.37 for the seeds of alfalfa, cucumber, radish, and tomato, respectively. The adsorption of NPs on seed coating is likely to cause toxicity to the growing vegetables.

### 3.3 Effect of NPs and BPs on relative seed germination

**Fig. 5:**
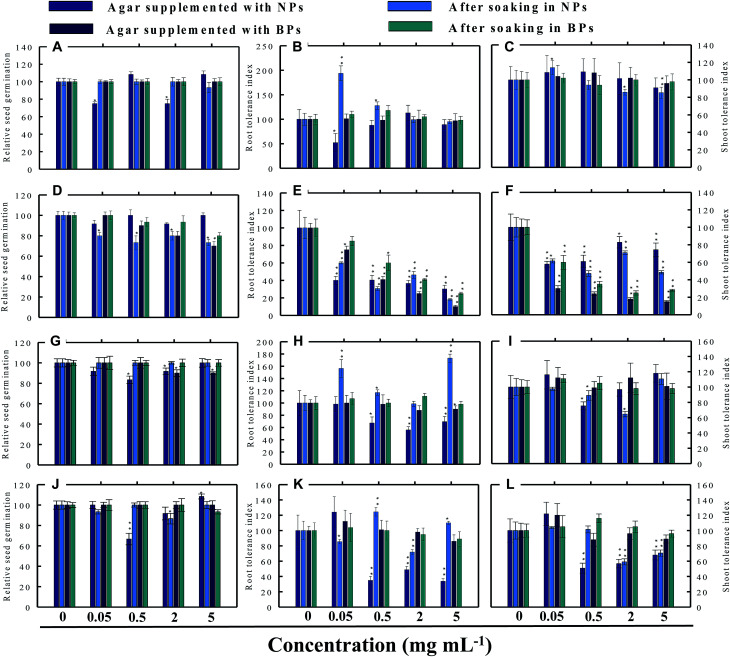
Dose–response effect of NPs and BPs of TiO_2_ (A), ZnO (B), Al_2_O_3_ (C), and CuO (D) before and after soaking (12 h) on relative seed germination (A, D, G and J), root tolerance index (B, E, H and K), and shoot tolerance index (C, F, I and L) of *R. sativus*. Values are given as mean ± S.D. of three independent replicates at *P ≤ 0.01, **P ≤ 0.05 *vs.* control.

**Fig. 6:**
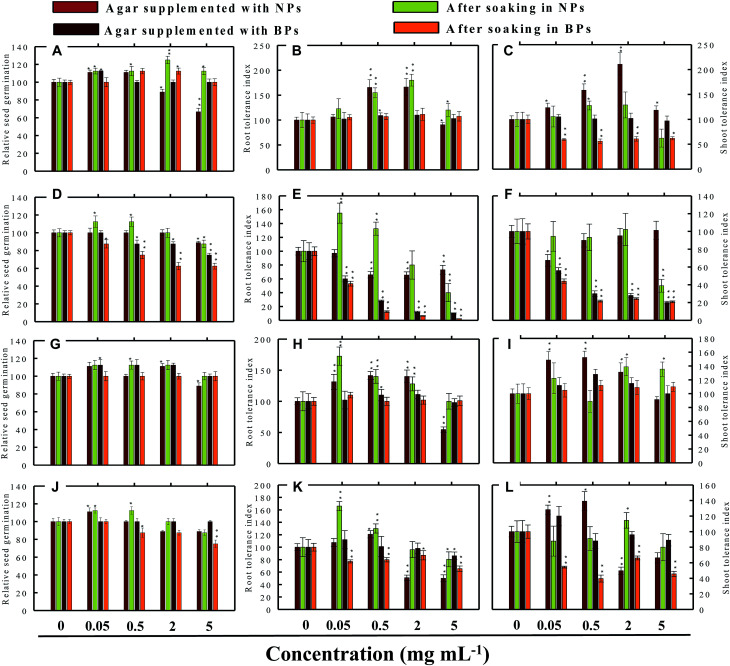
Dose–response effect of NPs and BPs of TiO_2_ (A), ZnO (B), Al_2_O_3_ (C), and CuO (D) before and after soaking (12 h) on relative seed germination, (A, D, G and J), root tolerance index (B, E, H and K), and shoot tolerance index (C, F, I and L) of *C. sativus*. Values are given as mean ± S.D. of three independent replicates at *P ≤ 0.01, **P ≤ 0.05 *vs.* control.

**Fig. 7:**
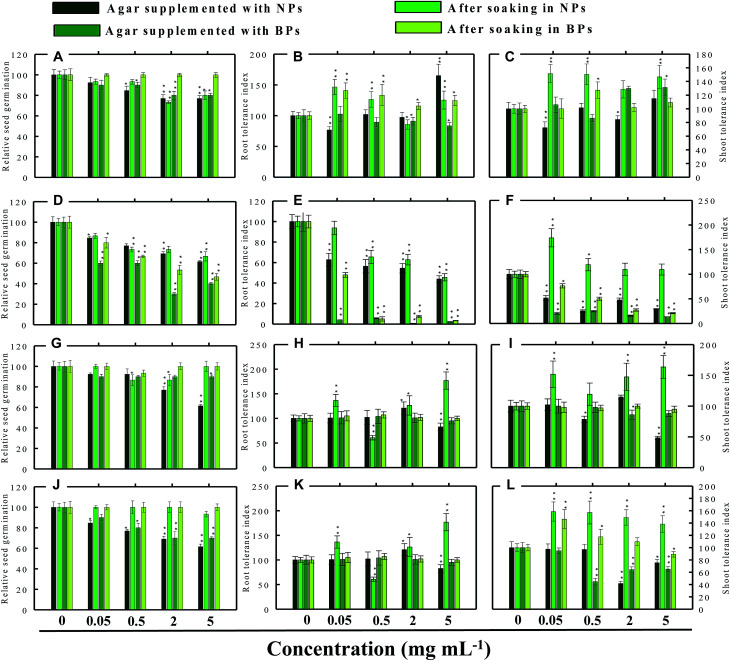
Dose–response effect of NPs and BPs of TiO_2_ (A), ZnO (B), Al_2_O_3_ (C), and CuO (D) before and after soaking (12 h) on relative seed germination, (A, D, G and j), root tolerance index (B, E, H and K), and shoot tolerance index (C, F, I and L) of *S. lycopersicon*. Values are given as mean ± S.D. of three independent replicates at *P ≤ 0.01, **P ≤ 0.05 *vs.* control.

**Fig. 8:**
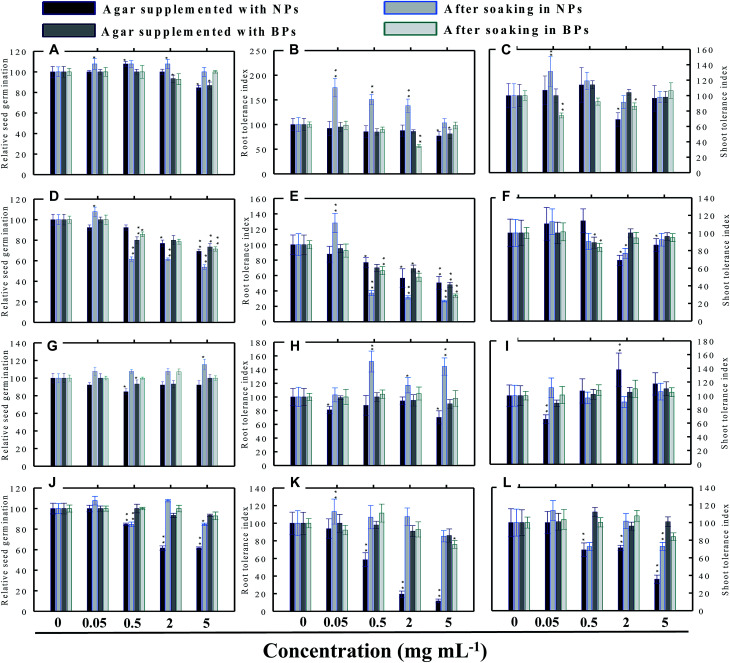
Dose–response effect of NPs and BPs of TiO_2_ (A), ZnO (B), Al_2_O_3_ (C), and CuO (D) before and after soaking (12 h) on relative seed germination, (A, D, G and J), root tolerance index (B, E, H and K), and shoot tolerance index (C, F, I and L) of *M. sativa*. Values are given as mean ± S.D. of three independent replicates at *P ≤ 0.01, **P ≤ 0.05 *vs.* control.

Seed germination is indeed the first stage toward the successful establishment of crops. The germination of seeds has therefore been widely used as an index to assay the phytotoxicity of agro-chemicals.^17^ Seed germination and root and shoot tolerance index of alfalfa, cucumber, radish and tomato, grown with and without NPs and BPs, varied considerably among the vegetables and under different MONP concentrations (Fig. 5–8). The impact of MONPs/BPs on four popularly grown vegetables is discussed in the following sections.

#### 3.3.1 Radish (*R. sativus*)

Generally, the RSG of the four vegetables declined gradually with consequent increase in the concentration of MONPs. For example, none of the MONPs exhibited any obvious toxic effects on the RSG of radish grown under both experimental setups (Fig. 5a, d, g and j). In contrast, on Hoagland's agar medium supplemented with 0.05 and 2 mg ml^−1^ TiO_2_-NPs, the RSG was significantly (*P* ≤ 0.01) reduced by 25% and 26%, respectively (Fig. 5a). Similarly, the aqueous dispersions of ZnO-NPs declined the RSG maximally by 27% at 5 mg ml^−1^ while ZnO-BPs inhibited the RSG maximally and significantly (*P* ≤ 0.01) by 30% at 5 mg ml^−1^ (Fig. 5d). The Al_2_O_3_-NPs at 0.5 and 2 mg ml^−1^ and Al_2_O_3_-BPs at 2 and 5 mg ml^−1^ reduced the RSG by 9–17% and 10%, respectively (Fig. 5g). A trend similar to TiO_2_-NPs was also obtained for CuO-NPs (Fig. 5j). None of the CuO-BPs concentrations, however, altered the RSG of radish.

#### 3.3.2 Cucumber (*C. sativus*)

In cucumber, raised on supplemented agar, TiO_2_-NPs at 0.05 mg ml^−1^ enhanced the RSG by 11% over control and after soaking with 2 mg ml^−1^, it enhanced the RSG maximally by 25% (Fig. 6a). On the contrary, 5 mg ml^−1^ TiO_2_-NPs reduced the RSG statistically (*P* ≤ 0.05) by 33% on supplemented agar medium (Fig. 6a). TiO_2_-BPs in contrast increased the RSG only at 2 mg ml^−1^ by 12% (Fig. 6a). Likewise, after soaking in ZnO-NPs (0.05 and 0.5 mg ml^−1^), the RSG was increased initially up to 12% but later it was reduced by 13% at 5 mg ml^−1^ (Fig. 6d). In contrast, ZnO-BPs reduced the RSG significantly (*P* ≤ 0.05) up to 25% on supplemented agar and up to 38% after soaking the seeds (Fig. 6d). None of the Al_2_O_3_-NPs or BPs concentrations altered the RSG of *C. sativus* except 5 mg ml^−1^ Al_2_O_3_-NPs, which reduced the RSG by 12% (Fig. 6g). Similarly, CuO-NPs did not show any negative impact on the RSG, while 5 mg ml^−1^ CuO-BPs reduced the RSG by 25% due to seed soaking (Fig. 6j).

#### 3.3.3 Tomato (*S. lycopersicon*)

TiO_2_-NPs in general were found inhibitory to tomato and reduced the RSG significantly (*P* ≤ 0.05) up to 24% when soaked on supplemented agar medium and 27% after soaking in NPs solution (Fig. 7a). The germination of seeds incubated on agar supplemented with TiO_2_-BPs was also reduced up to 20%, and hence both forms of ZnO were found significantly (*P* ≤ 0.05) inhibitory to the RSG of tomato. The ZnO-NPs and BPs reduced the RSG of tomato up to 39% and 60% (*P* ≤ 0.05), respectively, on supplemented agar media (Fig. 7d). While, after soaking in ZnO-NPs and BPs, the RSG was limited up to 35% and 54% (*P* ≤ 0.05), respectively (Fig. 7d). The inhibition of RSG by Al_2_O_3_-NPs was maximum at 5 mg ml^−1^ (39%) when seeds were grown on NPs-supplemented agar medium (Fig. 7g). The reduced RSG of tomato also reduced substantially when plants were grown on agar medium treated with different concentrations of CuO-NPs and BPs (Fig. 7j).

#### 3.3.4 Alfalfa (*M. sativa*)

As for the RSG of *M. sativa*, only TiO_2_-NPs and BPs reduced germination by 16% and 14%, respectively, at only 5 mg ml^−1^ (Fig. 8a). At 0.05 and 2 mg ml^−1^ concentration, the RSG of seeds soaked in TiO_2_-NPs increased up to 7%. Rest of the concentrations of NPs and BPs were found to be passive on the RSG of *M. sativa* (Fig. 8a). Both ZnO-NPs and ZnO-BPs at 5 mg ml^−1^ had maximum inhibitory effect on the RSG of *M. sativa* (Fig. 8d). In contrast, Al_2_O_3_-NPs at 5 mg ml^−1^, caused 15% increase in RSG (Fig. 8g) but other doses of Al_2_O_3_ were found to be ineffective. Contrarily, Al_2_O_3_-NPs and CuO-NPs declined the RSG up to 39% when raised on supplemented agar media and by 16% after dipping the seeds in CuO-NPs (Fig. 8j).

In support of our findings, alterations in seed germination of various plant species by metal and metal oxide NPs have been reported.^4,18^ For instance, nano forms of NiO, CuO, TiO_2_, Co_3_O_4_, and Fe_2_O_3_ have shown variable inhibitory effects on seed germination in radish, cucumber, and lettuce, which, however, differed with crop genotypes and seed size and followed the order: lettuce > cucumber > radish.^19^ In a similar study, López-Moreno *et al.* reported a substantial reduction in the germination of tomato (30%), cucumber (20%), and maize (30%) when exposed to cerium dioxide (CeO_2_) NPs at 2000 mg l^−1^ while germination of alfalfa plant remained unaffected.^20^

In contrast, the application of SiO_2_ NPs even at 8 g l^−1^ dose rate did improve seed germination, seed vigor index, and the average germination time of tomato.^21^ Yet, some other studies have shown variable impact of TiO_2_-NPs on seed germination of maize, radish, rapeseed, wheat, onion, tomato, fennel, and parsley.^4,22,23^ It has been reported that lower concentrations of NPs such as 10–40 ppm may enhance seed germination and seedling growth; however, concentrations >50 ppm have been found to exert a toxic impact on germination and seedling growth.^18,24^ Despite increase or decrease, it has been reported that aqueous TiO_2_-NP suspensions (14–25 nm) did not affect germination in rapeseed and wheat up to 100 ppm concentration.^25^ Recently, the treatment of rice seeds with 100–1000 ppm of TiO_2_- and ZnO-NPs was found to be passive for seed germination even after soaking the seeds for three days.^26^ In another study, 100% seed germination was recorded upon exposure of tomato and onion seeds to 100 mg ml^−1^ TiO_2_-NPs, while radish seeds were able to germinate by 100% at 400 mg ml^−1^.^27^ More recently, ZnO-NPs were found inert on seed germination of *Zea mays* and *C. sativus*.^28^

Despite conflicting reports on the effect of NPs on the germination efficiency of many plants, the current findings clearly suggest that lower concentrations of NPs may serve as seed-priming agents, but at higher concentrations the same NPs have deleterious impact on RSG. Among metal NPs, those prepared from Zn were found more inhibitory to all vegetable species.

### 3.4 Root and shoot development of vegetables under stress

The impact of NPs and BPs on root (RTI) and shoot growth (STI) of radish, cucumber, tomato and alfalfa varied with dose rates of both NPs and BPs (Fig. 5–8). The findings observed in this study are explained in the following sections.

#### 3.4.1 Effect of TiO_2_-NPs and BPs

The TiO_2_-NPs and BPs as a whole did not reduce the growth of the four vegetables significantly. Instead, some TiO_2_-NPs and BPs facilitated the growth of both root and shoot until certain concentrations. For instance, 0.05 mg ml^−1^ TiO_2_-NPs maximally increased the RTI of *R. sativus* by 93% after soaking (Fig. 5b), whereas, the STI was enhanced by 13% (Fig. 5c). Similarly, TiO_2_-NPs produced a dose-dependent increase in the RTI of *C. sativus* up to 66% (on supplemented agar) and 80% (after soaking) (Fig. 6b). The STI of *C. sativus* was augmented up to 110% (on supplemented agar) and 28% (after soaking) (Fig. 6c). After soaking in TiO_2_-BPs, the STI of *C. sativus*, in contrast, was inhibited up to 39% but remained constant (44–39%) up to 5 mg ml^−1^ (Fig. 6c). Similar increase in RTI and STI was observed for *S. lycopersicon* (Fig. 7b and c) and *M. sativa* (Fig. 8b and c). Root enhancement by TiO_2_-NPs in *S. lycopersicon* was up to 65% (on supplemented agar) and 25% (after soaking) (Fig. 7b), while, the maximum increment in shoot length was 14% (on supplemented agar) and 50% (after soaking) (Fig. 7c). Interestingly, after soaking in TiO_2_-BPs, a maximum increase of 24% in root length was also noticed (Fig. 7b). After soaking in 0.05 mg ml^−1^ of TiO_2_-NPs, the RTI of *M. sativa* was enhanced significantly (*P* ≤ 0.05) by 74%, which decreased thereafter in a concentration-dependent fashion (Fig. 8b). Except for 44% (*P* ≤ 0.05) reduction in root length on supplemented agar, no significant effects on root and shoot elongation was observed for other concentrations of TiO_2_-BPs.

#### 3.4.2 Effect of ZnO-NPs and BPs

In general, the ZnO-NPs and BPs displayed severe toxic impact on root and shoot elongation of all test vegetables, which however, differed with the concentration of each NP and BP. The maximum inhibition of the RTI of *R. sativus* was 75% (ZnO-NPs + agar), 82% (after soaking in ZnO-NPs), 90% (ZnO-BPs + agar), and 75% (after soaking in ZnO-BPs) (Fig. 5e). Likewise, maximum reduction in STI was found as 26% (ZnO-NPs + agar), 52% (after soaking in ZnO-NPs), 85% (ZnO-BPs + agar), and 72% (after soaking in ZnO-BPs) (Fig. 5f). The 50% inhibitory concentration (IC_50_) was defined in the current study as the concentration of NPs or BPs at which the root or shoot length shows a 50% decrease *vs.* the untreated control (100%). The IC_50_ of ZnO-NPs for *R. sativus* roots was found to be 0.05 (ZnO-NPs + agar) and 0.5 mg ml^−1^ (after soaking in ZnO-NPs). The BP of ZnO was also detrimental to the roots and shoots of *R. sativus*. The IC_50_ on supplemented agar treatments was 0.5 mg ml^−1^ for roots and 0.05 mg ml^−1^ for shoots, whereas, it was 2 mg ml^−1^ for roots and 0.5 mg ml^−1^ for shoots emerged from seeds after soaking in ZnO-BPs. Moreover, a 90% inhibitory concentration (IC_90_) was also observed for roots of *R. sativus* grown on ZnO-BPs-supplemented agar. For shoots of *C. sativus*, the IC_50_ for ZnO-NPs was found to be 5 mg ml^−1^ after soaking, while for ZnO-BPs, it was 0.5 mg ml^−1^ for both roots and shoots developed on supplemented agar and 0.5 and 0.05 mg ml^−1^ for roots and shoots after soaking. After a significant increase in root length (55%) at 0.05 mg ml^−1^ of ZnO-NPs (after soaking), it decreased in a dose-dependent manner and was found to be maximum (60%) at 5 mg ml^−1^ (Fig. 6e). Similarly, BPs also decreased root and shoot elongation in a dose-dependent manner with IC_90_ of 5 mg ml^−1^ (on supplemented agar) and 2 mg ml^−1^ (after soaking) (Fig. 6e). The root and shoot length of *S. lycopersicon* also decreased significantly (*P* ≤ 0.05) with increase in concentration of ZnO-NPs and BPs. IC_50_ for different treatments followed the order: 5 mg ZnO-NPs ml^−1^ for roots and 0.5 mg ZnO-NPs ml^−1^ for shoots grown on supplemented agar, 5 mg ZnO-NPs ml^−1^ for roots grown after soaking the seeds, 0.05 mg ZnO-BPs ml^−1^ for shoots grown on supplemented agar, and 0.05 mg ZnO-BPs ml^−1^ for roots and 2 mg ml^−1^ for shoots grown after soaking treatment. The IC_90_ calculated for ZnO-BPs was 0.05 mg ml^−1^ for roots on supplemented agar and 0.5 mg ml^−1^ for shoots after soaking. Beside the growth-inhibitory effects of ZnO-NPs on *S. lycopersicon*, a significant enhancement in shoot height was also observed for all concentrations of ZnO-NPs due to soaking (Fig. 7f). Among all plants, shoot growth of *M. sativa* was least affected by ZnO-NP and BP treatments (Fig. 8f); whereas, root elongation decreased with increase in concentration (Fig. 8e). The lowest concentration of ZnO-NPs (0.05 mg ml^−1^) showed 28% increase in root elongation (Fig. 8e). IC_50_ for *M. sativa* roots grown before and after soaking in ZnO-NPs was found to be 5 and 0.5 mg ml^−1^, whereas, for ZnO-BPs, it was 5 mg ml^−1^ for both treatments.

#### 3.4.3 Effect of Al_2_O_3_-NPs and BPs

The Al_2_O_3_-NPs decreased the RTI of *R. sativus* up to 45% in a concentration-dependent manner on supplemented agar media, whereas, 5 mg ml^−1^ showed 73% increase after soaking (Fig. 5h). For *C. sativus*, only 5 mg ml^−1^ of Al_2_O_3_-NPs decreased RTI and STI by 46% while all other concentrations enhanced RTI and STI from 10% to 72% (Fig. 6h and i). Likewise, only 5 mg Al_2_O_3_-NPs ml^−1^ decreased RTI and STI of *S. lycopersicon* by 18% and 52%, respectively, on supplemented agar (Fig. 7h and i). The RTI was promoted from 20%–76% on exposure to Al_2_O_3_-NPs and BPs at various concentrations (Fig. 7h), while enhancement in STI varied between 14% and 63% (Fig. 7i). The Al_2_O_3_-NPs and BPs, however, showed poor inhibitory effect on *M. sativa* (Fig. 8h and i). As an example, Al_2_O_3_-NPs at 5 mg ml^−1^, increased the RTI by 44% (Fig. 8h), while STI was found highest (37%) at 2 mg Al_2_O_3_-NPs ml^−1^ (Fig. 8i).

#### 3.4.4 Effect of CuO-NPs and BPs

A significant negative influence on RTI and STI of plants grown under CuO-NPs and BPs was observed. The CuO-NPs significantly (*P* ≤ 0.05) decreased RTI and STI of *R. sativus* from 15%–67% (Fig. 5k and l). Similar toxic effects were also observed for cucumber (Fig. 6k and 1), tomato (Fig. 7k and 1) and alfalfa (Fig. 8k and l). Besides exhibiting an inhibitory impact, some concentrations of CuO-NPs and BPs also showed growth-promoting effects, and, hence, enhanced the length of roots (RTI) and shoots (STI) of vegetables.

In our study, heavy MONPs exhibited dual characteristics, which has been reported only in few studies. In literature, the negative effects of metal-based NPs like ZnO, Al_2_O_3_, CuO, and TiO_2_ on shoot/root elongation and growth have been reported in various cereals (wheat, rice, maize, and barley) and certain vegetables (for example, tomato). The increased toxicity of NPs has been suggested to be due to the interaction that occurs between the NPs and plant exudates and the consequent release of metal ions from the NPs in growth media.^29,30^ The phytotoxic effect of Al_2_O_3_-NPs is largely considered to be inhibitory or neutral on plant growth.^31,32^ Therefore, their positive effect on root and shoot elongation is unexpected.

Even though the factors affecting the enhancement in root elongation following BP exposure are unclear, it might be presumed that since the agar matrix is nonporous the BPs are possibly trapped inside the polymeric network of polysaccharides (agar), and, therefore, the entry of BPs within the tissues of roots/shoots is restricted. On the other hand, nano forms of metals, for example TiO_2_-NPs and nanowires, have shown to significantly enhance root elongation and seed germination of wheat and lettuce as compared to bulk materials.^22,33^ This type of growth promotion also depends on – (i) the concentration and duration of NP exposure (ii) growth environment and (iii) plant species.^4,34^ In line with our study, lower range of ∼20 nm-sized TiO_2_-NPs (10–100 ppm) significantly increased root and shoot fresh biomass, whereas concentration >100 ppm was detrimental.^33^ Surprisingly, in another work, even 1000–5000 ppm of TiO_2_-NPs did not significantly change the biomass of tomato.^35^ Fragmentation of cellular DNA was also observed due to the toxicity of ZnO-NPs in onion.^36^ The exposure of wheat to CuO-NPs showed 5% and 13% decrease in root and shoot lengths, respectively, and exhibited necrosis in roots which as a result were thinner and more brittle compared to the control.^37^ Among the bulk materials tested, ZnO-BPs were also found toxic to seedlings specifically of *C. sativus* and *S. lycopersicon*. This could be due to following reasons – (i) chemical: release of Zn^2+^ ions from ZnO-NPs in growth medium followed by their uptake by seeds and seedlings above threshold level and (ii) physical intervention of ZnO-BPs with plant roots which might have blocked the water transport. NPs as a whole, may interfere with plant metabolism in several ways, such as by providing micronutrients, down and up regulation of genes, and interfering with oxidative processes, which result in oxidative burst.^30,38,39^ The entered nanoparticles can also interfere with the electron transport chain of mitochondria and chloroplast.^1^

### 3.5 NPs uptake by seeds and seedlings

**Fig. 9:**
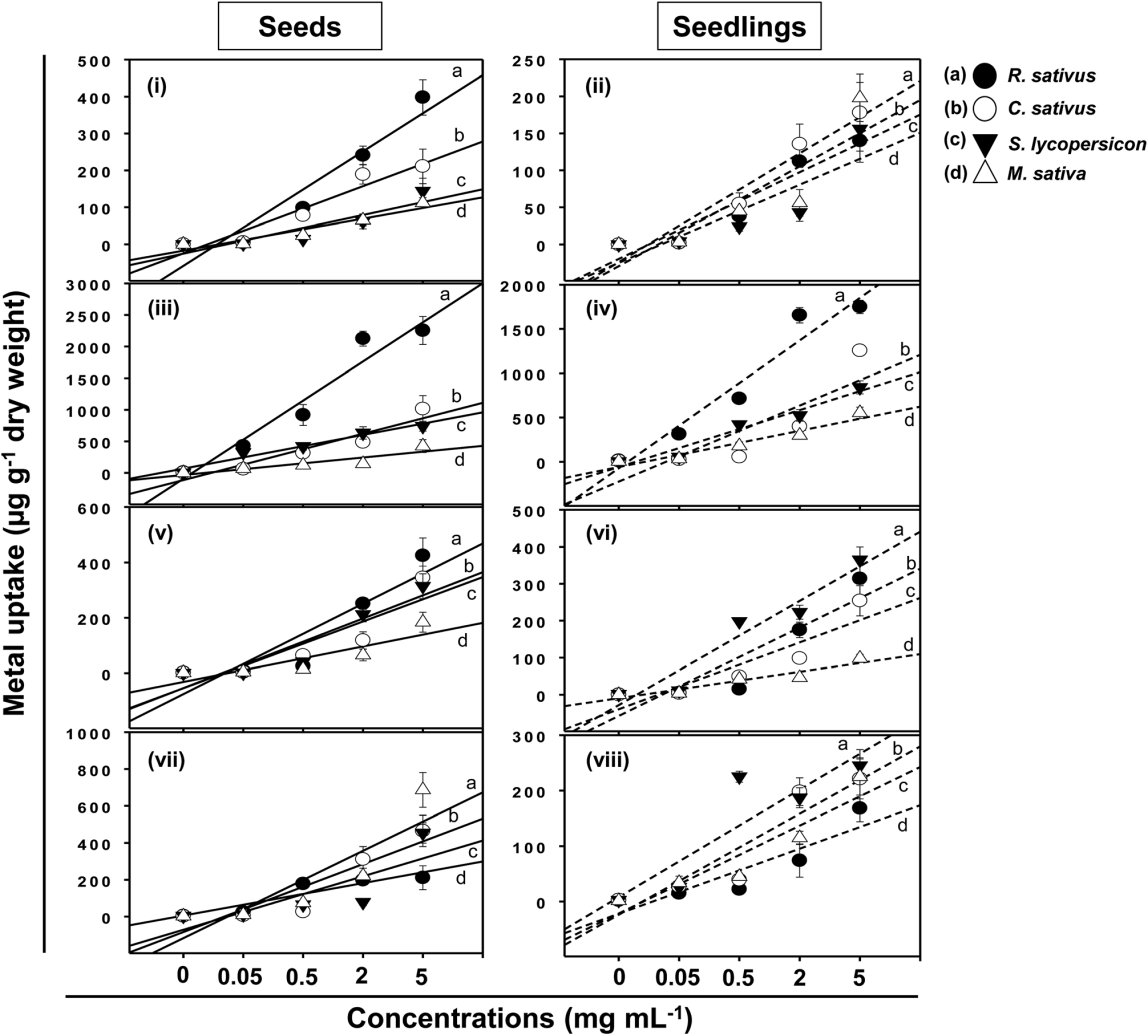
Linear line and scatter plots showing concentration-dependent uptake of heavy MONPs in seeds and seedlings exposed to 0.05–5 mg ml^−1^ NPs of TiO_2_ (i and ii), ZnO (iii and iv), Al_2_O_3_ (v and vi), and CuO (vii and viii). Letters a, b, c, and d represent *R. sativus*, *C. sativus*, *S. lycopersicon*, and *M. sativa*, respectively. Error bars stand for experiments performed in triplicates (*n* = 3).

Following adsorption onto seeds and plant surfaces, TiO_2_-NPs, ZnO-NPs, Al_2_O_3_-NPs, and CuO-NPs entered inside plant tissues and growing seedlings of test crops (Fig. 9). The concentration of MONPs, however, differed in a dose-dependent manner and varied greatly between seeds and seedlings of each plant. For example, the content of Ti accumulated within the seeds of *R. sativus* growing at 0.05–5 mg Ti ml^−1^ ranged between 4 and 398 μg g^−1^ while it varied between 2 to 140 μg g^−1^ in seedlings. Likewise, the concentration of Ti in seeds/seedlings of *C. sativus*, *S. lycopersicon* and *M. sativa* was recorded as (μg g^−1^): 211/178, 144/156, and 112/198, respectively (Fig. 9i and ii). Similarly, the ZnO-NPs were found maximum in seeds/seedlings of *R. sativus* among all test plants (2254/1748 μg g^−1^) which was followed by *C. sativus* (1015/1254 μg g^−1^), *S. lycopersicon* (741/850 μg g^−1^) and *M. sativa* (425/554 μg g^−1^) (Fig. 9iii and iv). The uptake of Al_2_O_3_-NPs (Fig. 9v and vi) and CuO-NPs (Fig. 9vii and viii) in seeds/seedlings of *R. sativus*, *C. sativus*, *S. lycopersicon*, and *M. sativa* followed a trend similar to those recorded for other MONPs. Summarily, the uptake pattern of heavy MONPs in seeds (A) and seedlings (B) of different crops followed the order:

A: (i) TiO_2_-NPs – *R. sativus* > *C. sativus* > *S. lycopersicon* > *M. sativa*; (ii) ZnO-NPs – *R. sativus* > *C. sativus* > *S. lycopersicon* > *M. sativa*; (iii) Al_2_O_3_-NPs – *R. sativus* > *C. sativus* > *S. lycopersicon* > *M. sativa*; (iv) CuO-NPs – *M. sativa* > *C. sativus* > *S. lycopersicon* > *R. sativus*.

B: (i) TiO_2_-NPs – *M. sativa* > *C. sativus* > *S. lycopersicon* > *R. sativus*; (ii) ZnO-NPs – *R. sativus* > *C. sativus* > *S. lycopersicon* > *M. sativa*; (iii) Al_2_O_3_-NPs – *S. lycopersicon* > *R. sativus* > *C. sativus* > *M. sativa*; (iv) CuO-NPs – *S. lycopersicon* > *M. sativa* > *C. sativus* > *R. sativus*.

An increase from 2 to 5 mg ml^−1^ of MONPs did not show any significant enhancement in metal uptake by seeds except for the ZnO-NP uptake by *M. sativa* and *C. sativus*. Similar observations were made in case of metal uptake in seedlings again with the exception of ZnO-NP uptake in *C. sativus*, *S. lycopersicon*, and *M. sativa*. This variation in MONP uptake could possibly be due to the differences in the aggregate-forming ability of NPs.^40^ However, despite variations, similar accumulation of MONPs has been reported in many plants. In a study, Al_2_O_3_-NPs at 1000 mg l^−1^ resulted in 350.5 μg g^−1^ accumulation of Al in plants, which did not cause root-growth inhibition.^13^ This could be explained to be due to the compartmentalization of inert MONPs in various plant cell organelles.^41,42^ Moreover, a limited uptake of TiO_2_-NPs by lettuce occurred due to the aggregation of NPs to much larger sizes after being mixed with the cultivation media.^33^ It has been suggested that the higher content of metal NPs in seedlings is a result of extensive attachment of particles on plant surfaces.^13^ Suriyaprabha *et al.* reported the bioaccumulation of transition metal oxide (Fe_2_O_3_, CuO and ZnO) NPs and their influence on *Vigna unguiculata* seeds.^43^ Apart from these, MONPs, for example CuO-NPs, are reported to induce DNA damage besides altering the structure of plant roots.^44,45^ The accumulation of MONPs within plant tissues, however, poses a serious and unexpected threat to human health which requires urgent attention.

### 3.6 Production of metallothioneins

**Fig. 10:**
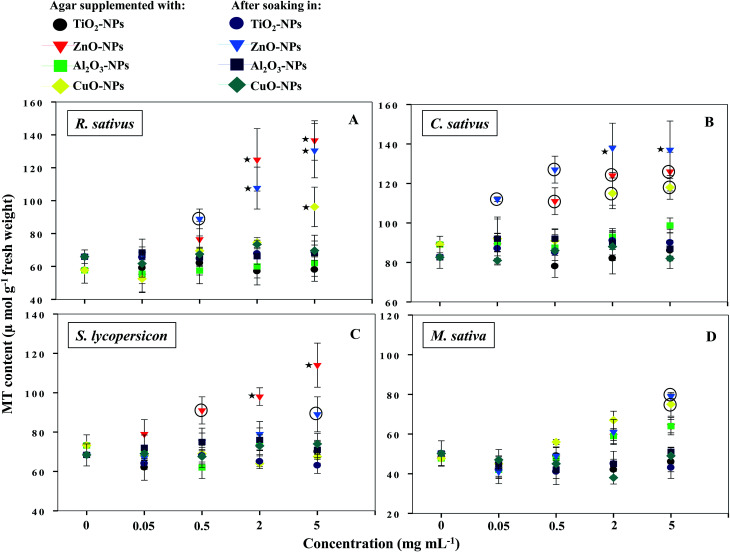
Dot plot of spectrophotometric detection of MTs in TiO_2_-, ZnO-, Al_2_O_3_-, and CuO-NP-treated *R. sativus* (A), *C. sativus* (B), *S. lycopersicon* (C), and *M. sativa* (D). Values are given as mean ± S.D. of experiments performed in triplicates (*n* = 3) with 10 seeds per replicate. Dots encircled and marked with star represent *P* ≤ 0.01 and *P* ≤ 0.05 *vs.* control, respectively.

**Fig. 11:**
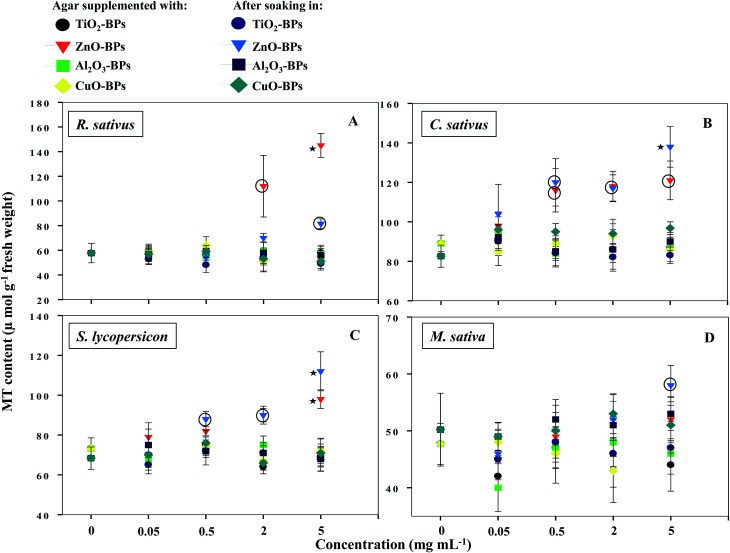
Dot plot of spectrophotometric detection of MTs in TiO_2_-, ZnO-, Al_2_O_3_-, and CuO-BP-treated *R. sativus* (A), *C. sativus* (B), *S. lycopersicon* (C), and *M. sativa* (D). Values are given as mean ± S.D. of experiments performed in triplicates (*n* = 3) with 10 seeds per replicate. Dots encircled and marked with star represent *P* ≤ 0.01 and *P* ≤ 0.05 *vs.* control, respectively.

NPs that once enter the plant tissues, are likely to be transformed, which can be mediated by cellular metabolic processes.^1^ During such processes, heavy metal ions are released from NPs, which in turn induce the generation of ROS and cause membrane lipid peroxidation.^5,14^ In contrast, plants have evolved certain mechanisms like the synthesis of metallothioneins (MTs) to hammer out metal toxicity; the binding affinity of MTs, however, varies with metal species.^46^ To the best of our information, the generation of MTs has yet not been reported as a phytotoxicity endpoint of NPs. Hence, the release of MTs by plants after soaking seeds in various concentrations of MONPs and BPs and growing them on supplemented semi-solid agar plates was detected and measured (Fig. 10a–d and Fig. 11a–d). Roots of untreated *R. sativus*, *C. sativus*, *S. lycopersicon*, and *M. sativa* secreted 252, 205, 314, and 88 μmol MTs g^−1^ fresh weight (FW). Among the species of metals used in this study, only bulk and NPs of ZnO and CuO were found stimulatory for MTs while MTs produced under exposure to TiO_2_- and Al_2_O_3_-NPs and BPs were statistically insignificant. MT production under ZnO-NPs and BPs followed a dose-dependent increase (Fig. 10 and 11). At 5 mg ml^−1^ ZnO-NPs, MT production in *R. sativus* was significantly high (136 μmol MTs g^−1^ FW) as compared to the negative control (57 μmol MTs g^−1^ FW) when grown on supplemented agar (Fig. 10a). Similarly, soaking of seeds in 0.5, 2, and 5 mg ml^−1^ ZnO-NPs resulted in the significant production of MTs: 85, 110 and 140 μmol MTs g^−1^ FW, respectively. Of the different CuO-NP concentrations, only 5 mg ml^−1^ of CuO-NPs exhibited significant (*P* ≤ 0.05) production of MTs (96.2 μmol MTs g^−1^ FW) (Fig. 10a). *C. sativus* on agar supplemented with ZnO-NPs and after soaking in ZnO-NPs showed MT production in a dose-related fashion and was found up to 126 and 137 μmol g^−1^ FW, respectively (Fig. 10b). While, CuO-NPs increased MTs maximally by 118 μmol g^−1^ FW compared to the untreated control (89 μmol g^−1^ FW). ZnO-NPs-amended agar also induced MT production in *S. lycopersicon* up to 114 μmol g^−1^ FW, whereas, soaking treatment increased MTs up to 89 μmol g^−1^ FW over the untreated control (Fig. 10c). *M. sativa* seedlings treated with CuO-NPs and ZnO-NPs (5 mg ml^−1^) showed 75 and 79 μmol MTs g^−1^ FW (Fig. 10d). Moreover, ZnO-BP-treated *R. sativus*, *C. sativus*, *S. lycopersicon*, and *M. sativa* displayed 145, 121, 98, and 52 μmol MTs g^−1^ FW (on supplemented agar) and 81, 138, 112, and 58 μmol MTs g^−1^ FW (after soaking), respectively (Fig. 11a, b, c and d). The role of MTs has been proposed in ROS scavenging where bound metals are released from the MT molecule and in turn ROS species bind to the cysteine (Cys) residue of the same.^47^ For normal cellular functioning, Zn mobilization from one Zn binding site to another is required, which may either constitute a general pathway by which Zn is distributed in the cell or be restricted to tackle oxidative stress.^48^ It has been reported that as many as 18 different metals may associate with MTs.^46^ Heavy metals are known to generate oxidative stress, damage cellular membranes and DNA, and disrupt cellular homeostasis.^49^ In the first line of defense, plants have strategies that prevent or reduce the uptake of metal ions by the apoplast by binding them to the cell wall or to cellular exudates, or by inhibiting long-distance transport.^50^ In contrast, when present at elevated concentrations, cells activate a complex network of detoxification tactics, such as chelation of metal ions with MTs in the cytosol and vacuolar sequestration by vacuolar transporters called compartmentalization.^51^ High thermodynamic and low kinetic stability are the main features of the metal–MT complex lagging behind a part of the metal which can be exchanged for other proteins.^48^

In a study by Yang *et al.*, genes responsible for metal homeostasis were found to be up regulated in wheat shoots grown in the presence of ZnO- and CuO-NPs.^52^ These genes were reported for encoding proteins for metal chelating compounds that might be metallothionein, chemocyanin, and blue-copper-binding proteins. Additionally, a battery of genes for plant defense response was also up regulated.^52^ Based on the toxic impacts exerted by metal oxide species (NPs and BPs) on crop plants, the following sensitivity order is suggested; for *R. sativus*: ZnO-NPs > ZnO-BPs > CuO-NPs > Al_2_O_3_-NPs > TiO_2_-NPs > CuO-BPs > Al_2_O_3_-BPs ∼ TiO_2_-BPs; *C. sativus*: ZnO-BPs > ZnO-NPs > CuO-NPs > CuO-BPs > TiO_2_-NPs > Al_2_O_3_-NPs > TiO_2_-BPs ∼ Al_2_O_3_-BPs; *S. lycopersicon*: ZnO-BPs > ZnO-NPs > CuO-NPs > Al_2_O_3_-NPs > TiO_2_-NPs > CuO-BPs > Al_2_O_3_-BPs ∼ TiO_2_-BPs; and *M. sativa*: ZnO-NPs > CuO-NPs > ZnO-BPs > Al_2_O_3_-NPs > TiO_2_-NPs > TiO_2_-BPs ∼ CuO-BPs ∼ Al_2_O_3_-BPs.

## 4. Conclusion

In conclusion, heavy metal oxide NPs as a whole reduced the relative seed germination and root and shoot tolerance indices of plants. Adsorption of MONP aggregates on seeds was significant and followed a dose-dependent uptake in both sets of treatments. Heavy MONPs of TiO_2_, ZnO, Al_2_O_3_, and CuO formed agglomerates ranging from 148 to 248 nm in growth media. Plants' dual responses that varied among MONPs and BPs were correlated to the concentrations tested. Among all test species, bulk and NPs of ZnO were found extremely detrimental to the measured growth parameters. In contrast, certain concentrations, specifically those of TiO_2_ and Al_2_O_3_ NPs, facilitated root and shoot elongation. However, how MONPs cause the positive or negative influence is not very clearly understood yet. It can be presumed that the internalized NPs could have been compartmentalized in cellular organs after partial or no transformation in growth medium and intracellular environment. Alternatively, the enhanced gaseous exchange due to the amendment of NPs in compact agar media could promote root and shoot elongation. Nano and bulk species of ZnO and CuO were found stimulatory for metallothionein production. Generally, the response of crops to NPs/BPs varied with dose and plant genotypes. Conclusively, the present study demonstrates the possible toxic effects of submicron and nano forms of heavy metal oxides on edible crops, which warrants the need for safe and regulated disposal of industrial/domestic wastes containing heavy MONPs. Also, the use of Cu, Zn, Al, and Ti oxides requires careful monitoring before/after they are discharged into the agro-ecosystems. Henceforth, the quantitative estimation of NPs in edible crops should be considered cautiously before they are supplied to consumers in order to prevent human health problems.

## Conflicts of interest

The authors declare that there are no conflicts of financial or personal interest.

## Supplementary Material

RA-009-C8RA09305A-s001
