# Supplementary material for: Understanding the phyto-interaction of heavy metal oxide bulk and nanoparticles: evaluation of seed germination, growth, bioaccumulation, and metallothionein production
Source: RSC Adv. 2019 Feb 1;9(8):4210–25. doi: 10.1039/c8ra09305a (PMC9060428; doi:10.1039/c8ra09305a)
Supplement: RA-009-C8RA09305A-s001 [file RA-009-C8RA09305A-s001.pdf]

Electronic Supplementary Material (ESI) for RSC Advances.
This journal is © The Royal Society of Chemistry 2019

Electronic Supplementary Information (ESI)

Supplementary Methods

Characterization of bulk and nanoparticles

MONPs were characterized by scanning electron microscopy (SEM), transmission electron microscopy (TEM), atomic force microscopy (AFM), quasi-elastic light scattering (QLS), fourier transform infrared (FTIR) spectroscopy, energy dispersive X-ray (EDX), zeta potential analysis, and X-ray diffraction (XRD). FT-IR spectra of NPs were recorded by Perkin Elmer FT-IR spectrometer, Spectrum Two (Perkin Elmer Life and Analytical Sciences, CT, USA) in KBr phase (1:100). MONPs were measured at 4000-450 cm^{-1} by FTIR. XRD patterns of NPs were recorded by MiniFlexII Desktop X-ray diffractometer (Rigaku Corporation, Tokyo, Japan) at 30kV/15mA. Peak intensities were observed from 20º to 80º 2θ angles. Average particle size of NPs was calculated by Debye-Scherrer's equation. SEM analysis was done using fine powder of NPs on a carbon tape in Field Emission Scanning Electron Microscope with embedded EsB Detector (Carl Zeiss Ultra Plus) equipped with EDX spectrometer at an accelerating voltage of 15 kV. Transmission electron microscopy analysis of NPs and their bulk counterparts was carried out using JEOL 100/120 kV TEM (JEOL, Tokyo, Japan) operating at a voltage of 200 kV. Samples were prepared by drying 10µl of ultrasonicated NPs (at 40 % amplitude for 20 min) on a copper grid at room temperature. A total of 10 micrographs from each MONP grid were examined to determine the percent frequency of size distribution and average primary particle size. Suspension of 50 µgNPs ml^{-1} was prepared in Hoagland's solution and sonicated for 10 min. at 40% amplitude. The hydrodynamic size of the NPs in nutrient suspension was measured by ZetaSizer Nano-ZS90, Malvern, UK. The ζ-potential values of the NPs were determined and the values presented are the average of 10 readings. Bulk particles of CuO and TiO_2 were procured from HiMedia, Mumbai, India whereas bulk-ZnO and Al_2O_3 were obtained from Thermo Fisher Scientific Pvt. Ltd., Mumbai, India and Merck, Mumbai, India.

Adsorption of NPs on plant seeds:

Seeds were put on the two sided carbon tape fixed on SEM stub followed by gold sputter coating for 20 min. Samples were then visualized under JSM 6510LV SEM (JEOL, Tokyo, Japan) at an accelerating voltage of 15 kV from lower to higher magnifications. The adsorption of NPs on seed surface was detected by Oxford Instruments INCAx-sight EDX spectrometer.

Internalization of metal in plant seeds and seedlings

The dried seed and seedling biomass was ground to powder using mortar and pestle. The samples (0.5 g) were then acid digested with concentrated HNO_3 (10 ml) for 2 h followed by filtration through 0.45 micron membrane filter. The filtered samples were analyzed using Inductively Coupled Plasma Mass Spectrometry (ICP-MS; Perkin Elmer ELAN DRC-e, USA) to determine the concentration of Ti and Al, whereas, Zn and Cu were determined through double beam flame-atomic absorption spectrophotometer (AAS), GBC 932B plus, Australia.

Supplementary Figures:

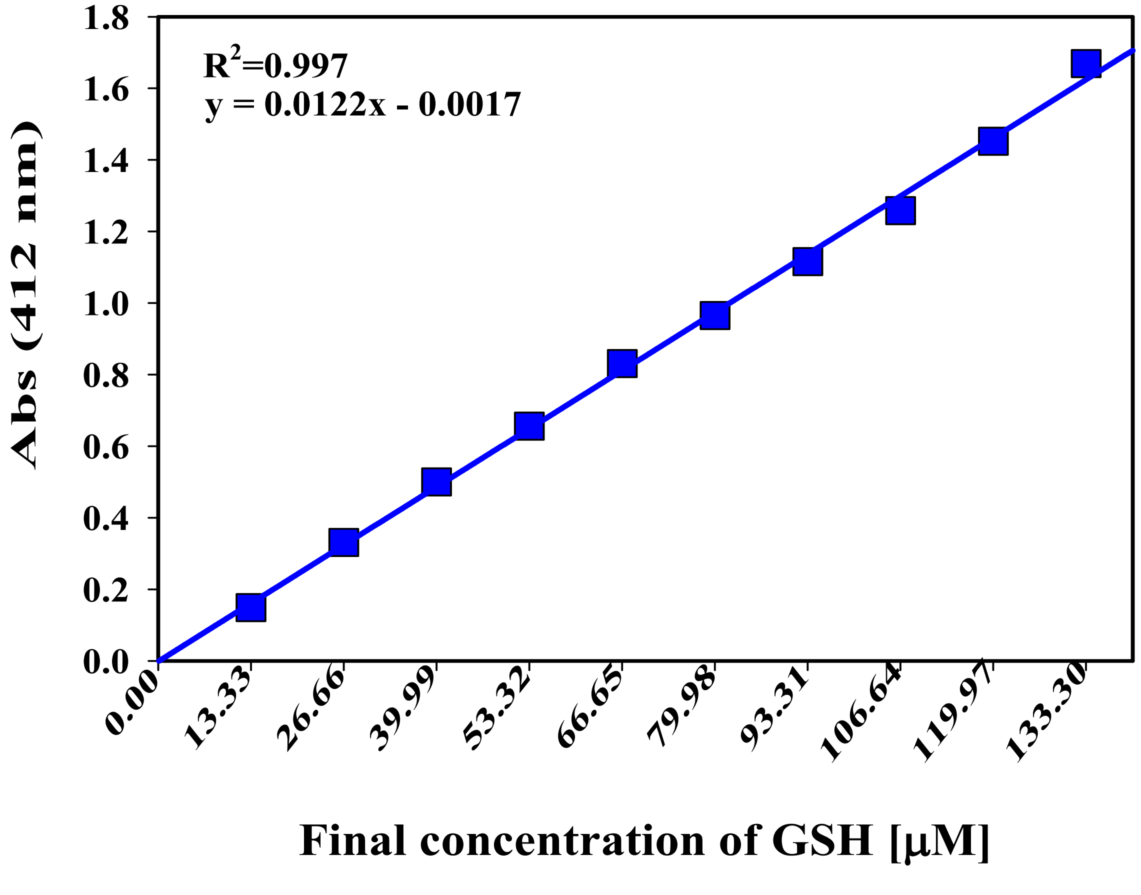

Supplementary Fig. 1 Standard curve of glutathione.

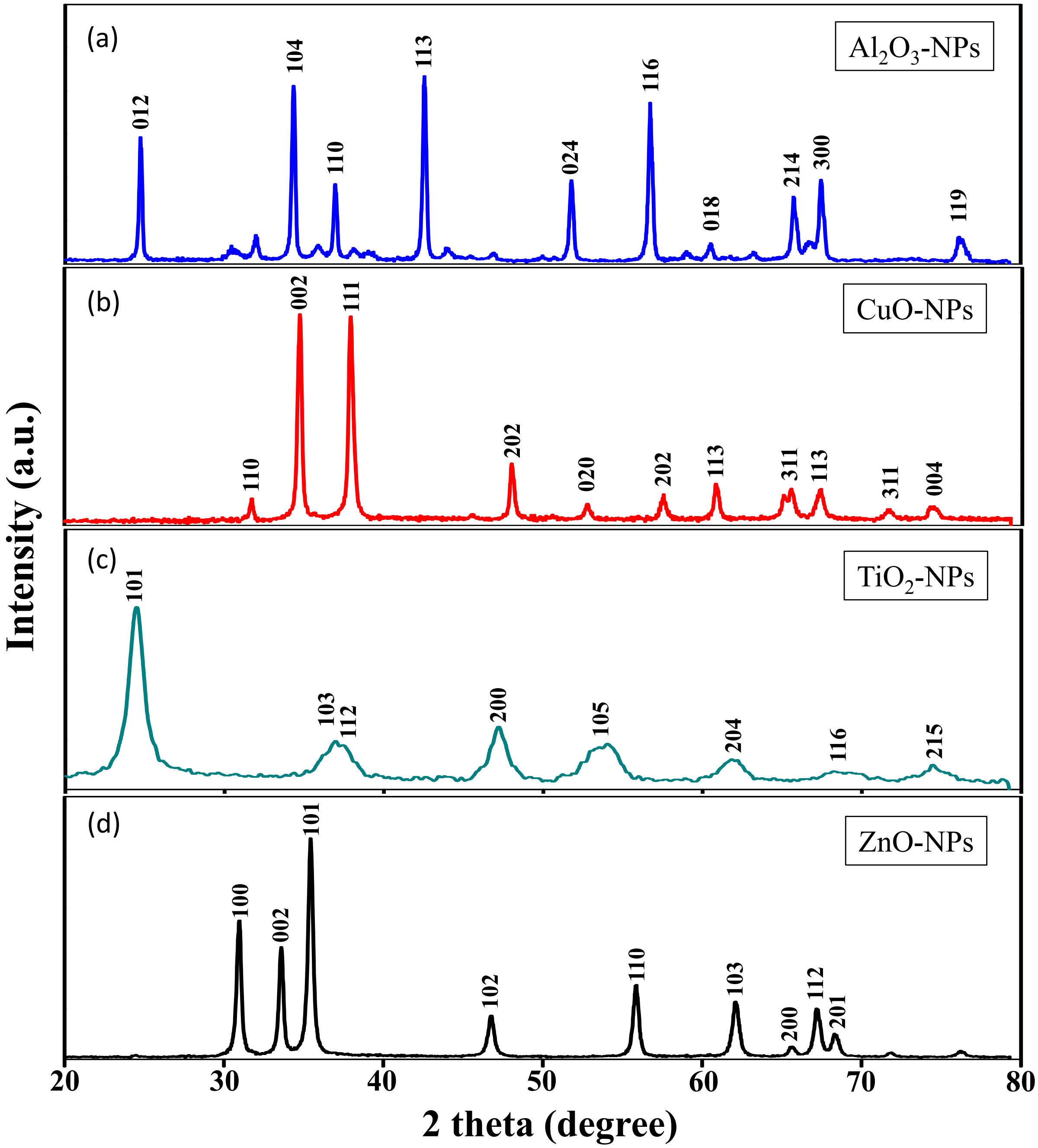

Supplementary Fig. 2 X-ray diffractograms of NPs recorded between 20-80 2θ° : Al2O3-NPs (a), CuO-NPs (b), TiO2-NPs (c), and ZnO-NPs (d).

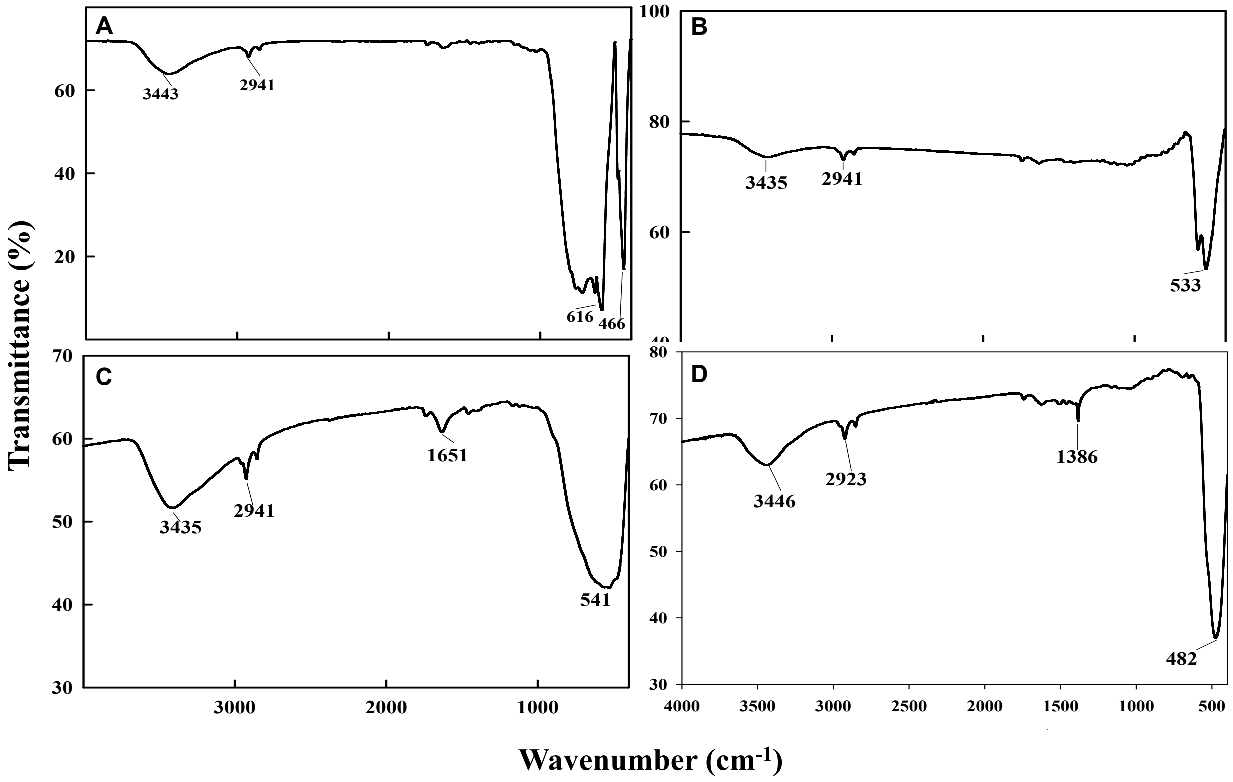

Supplementary Fig. 3 FTIR vibrational spectra of NPs: Al_2O_3-NPs (a), CuO-NPs (b), TiO_2-NPs (c), and ZnO-NPs (d) in the range of 400-4000 cm^{-1}.

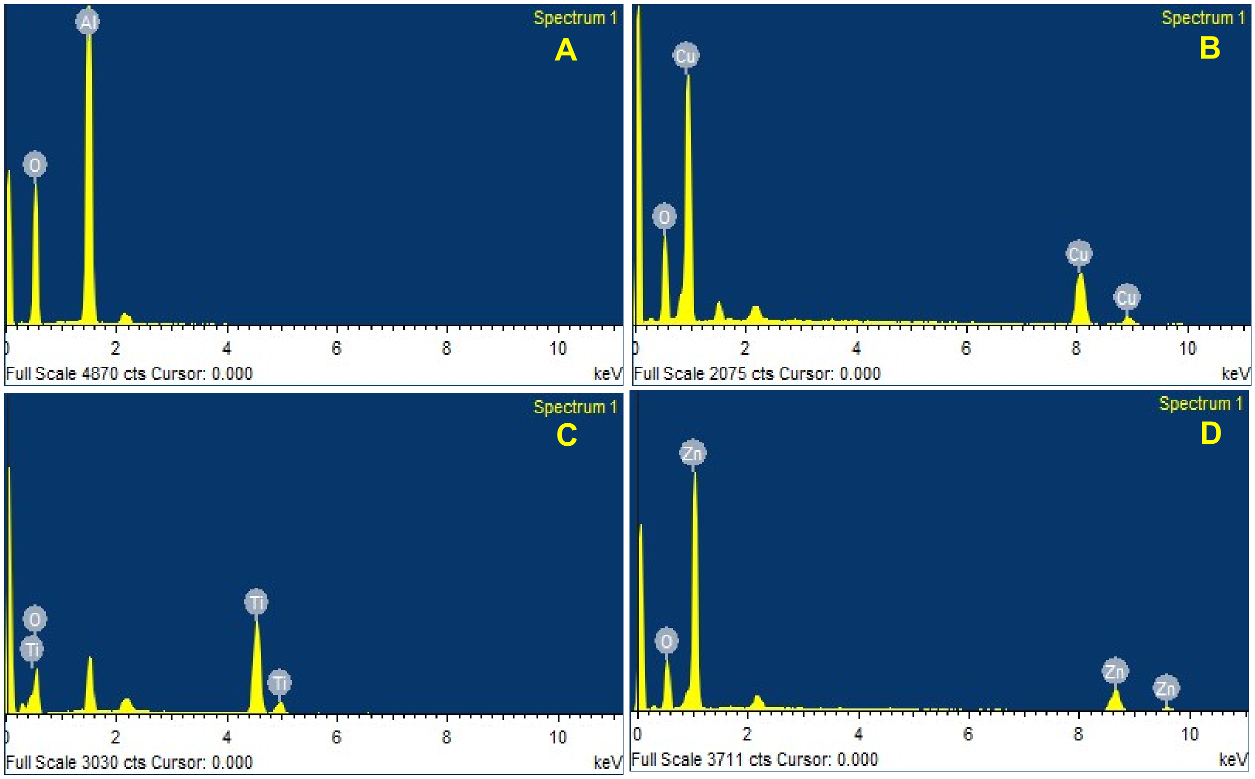

Supplementary Fig. 4 EDX spectra of NPs: Al_2O_3-NPs (a), CuO-NPs (b), TiO_2-NPs (c), and ZnO-NPs (d).

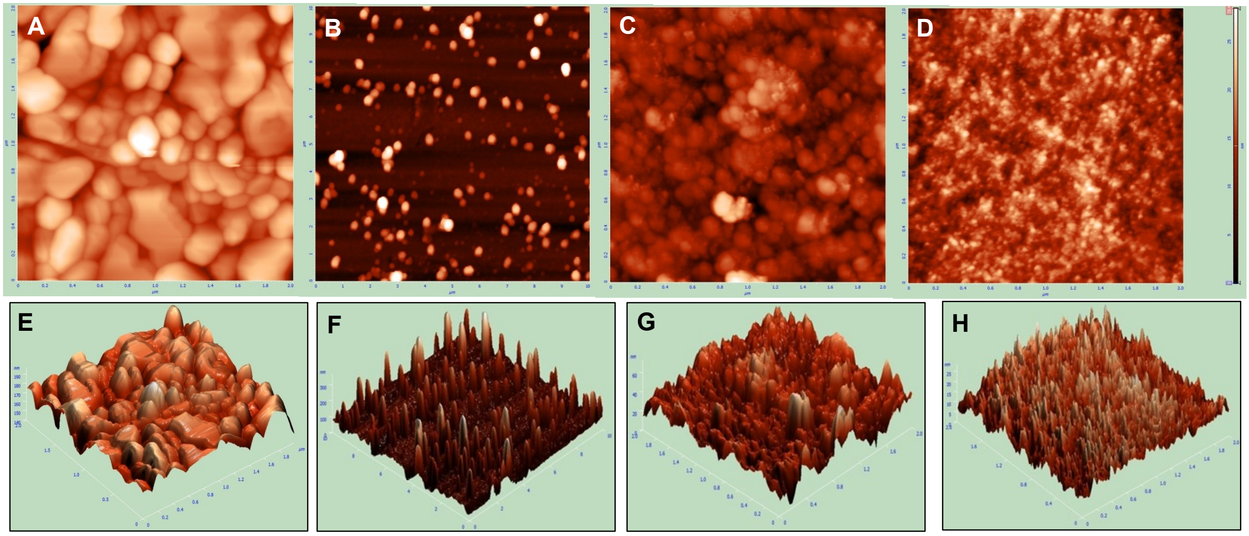

Supplementary Fig. 5 AFM 2D (a-d) and 3D (e-h) images of NPs: Al_2O_3-NPs (a, e), CuO-NPs (b, f), TiO_2-NPs (c, g), and ZnO-NPs (d, h).

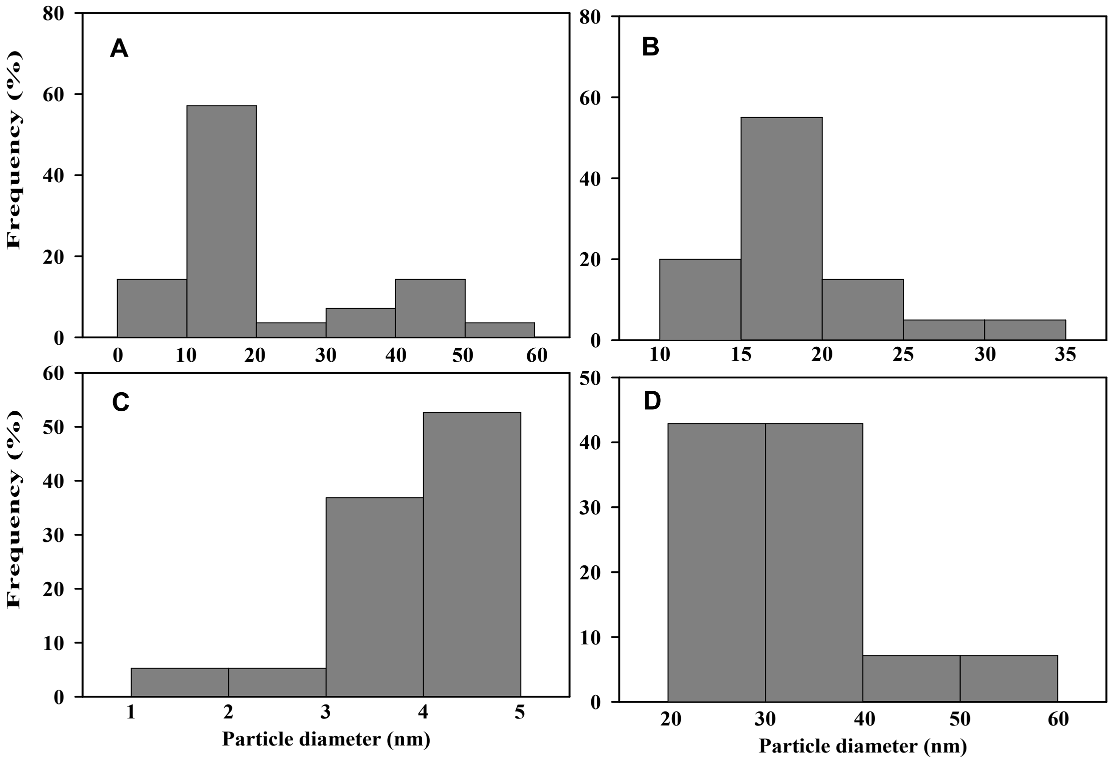

Supplementary Fig. 6 Frequency size distribution of NPs: Al_2O_3-NPs (a), CuO-NPs (b), TiO_2-NPs (c), and ZnO-NPs (d).
